# DNA Damage Responses in Tumors Are Not Proliferative Stimuli, but Rather They Are DNA Repair Actions Requiring Supportive Medical Care

**DOI:** 10.3390/cancers16081573

**Published:** 2024-04-19

**Authors:** Zsuzsanna Suba

**Affiliations:** Department of Molecular Pathology, National Institute of Oncology, Ráth György Str. 7-9, H-1122 Budapest, Hungary; subazdr@gmail.com; Tel.: +36-00-36-1-224-86-00; Fax: +36-00-36-1-224-86-20

**Keywords:** anti-estrogen, cancer therapy, estrogen, DNA damage, DNA damage response, DNA repair, endocrine disruptor, estrogen receptor, growth factor receptor, mutation

## Abstract

**Simple Summary:**

This work challenges the traditional principles of cancer therapy: simply targeting and blocking the regulatory pathways of rapidly proliferating tumors is a serious mistake. Since tumor initiation and growth may be attributed to a patient’s genomic instability and damage, genotoxic medications are inappropriate as they cause additional genomic damage in both patients and their cancers. Tumor cells are not enemies to be killed, but rather they are ill human cells which have the remnants of same genome stabilizer pathways like healthy cells. Within tumors, there is a combat for the improvement of their genomic defects. Moreover, tumors ask for help in their kamikaze action by recruiting immune competent cells into their environment. We should learn by watching the genome repairing activities within tumors, in the peritumoral region and in the whole body, and may follow them with supportive care. Successful cancer therapy does not remain a dream to be realized in the far future, but we should set about a cancer cure without delay.

**Abstract:**

Background: In tumors, somatic mutagenesis presumably drives the DNA damage response (DDR) via altered regulatory pathways, increasing genomic instability and proliferative activity. These considerations led to the standard therapeutic strategy against cancer: the disruption of mutation-activated DNA repair pathways of tumors.Purpose: Justifying that cancer cells are not enemies to be killed, but rather that they are ill human cells which have the remnants of physiologic regulatory pathways. Results: 1. Genomic instability and cancer development may be originated from a flaw in estrogen signaling rather than excessive estrogen signaling; 2. Healthy cells with genomic instability exhibit somatic mutations, helping DNA restitution; 3. Somatic mutations in tumor cells aim for the restoration of DNA damage, rather than further genomic derangement; 4. In tumors, estrogen signaling drives the pathways of DNA stabilization, leading to apoptotic death; 5. In peritumoral cellular infiltration, the genomic damage of the tumor induces inflammatory cytokine secretion and increased estrogen synthesis. In the inflammatory cells, an increased growth factor receptor (GFR) signaling confers the unliganded activation of estrogen receptors (ERs); 6. In breast cancer cells responsive to genotoxic therapy, constitutive mutations help the upregulation of estrogen signaling and consequential apoptosis. In breast tumors non-responsive to genotoxic therapy, the possibilities for ER activation via either liganded or unliganded pathways are exhausted, leading to farther genomic instability and unrestrained proliferation. Conclusions: Understanding the real character and behavior of human tumors at the molecular level suggests that we should learn the genome repairing methods of tumors and follow them by supportive therapy, rather than provoking additional genomic damages.

## 1. Introduction

Cancer is a complex disease, presumably originating from mutations in genes, promoting genomic instability, and initiating cancer development [1]. In cancers, mutagenesis drives the DNA damage response (DDR) via altered regulatory pathways, increasing genomic instability and helping proliferative activity [2]. Altered DNA damage responses in tumors serve the maintenance of survival and unrestrained proliferative activity of cells. These considerations led to the standard therapeutic strategy against cancer: the disruption of mutation-activated DNA repair pathways of tumors, which should lead to the clinical recovery of cancer patients [3]. However, the derangement of the mutation-driven DNA repair techniques of tumors could not bridge the gap between basic research and clinical practice.

In tumors, the accumulation of somatic mutations yields so-called cancer driver genes, and their altered regulatory protein products may manage aggressive expansion [4]. Catalogues of genes known to be involved in cancer development were prepared by whole-exome and later, whole-genome sequencing of numerous tumor samples. Analyses of thousands of cancer genomes return a remarkably similar catalogue of around 300 genes that are mutated in at least one cancer type. Yet, many features of these mutated genes and their exact role in cancer development remain unclear. The accumulation of certain mutated genes in tumors is not enough to justify their pro-oncogenic nature.

There is a close collaboration between the activity of the immune system and cancer driver mutations. The immune system has a strong impact on determining the expression of certain cancer driver genes [5]. At the same time, the appearance of certain cancer driver mutations shows correlations with the density and composition of immune competent cells in the tumor microenvironment [6]. The connection of the immune system with the appearance of cancer driver mutations is probably mediated by the fact that all somatic mutations can create neoantigens. These unknown peptides may trigger an immune response, eliminating the cell that carries them; this process is known as immune-editing [5].

Cancer driver mutations influence the quantity and composition of immune cell infiltration in the tumor microenvironment [6]. Somatic mutations in cancer driver genes with well-known roles in immune signaling, such as *CASP8* or *HLA*, generally recruit higher concentrations of immune cells into the tumor microenvironment. These pro-oncogenic mutations most likely result in immune-evading mechanisms. In contrast, colorectal tumors, with accumulated *KRAS* mutation, show weaker immune cell infiltration than those without this mutation, and the tumors are resistant to the immune-checkpoint blockade [7].

Surprisingly, cancer driver genes are exposed, even in various healthy cells exhibiting the same somatic mutations as tumors. Two studies examined somatic mutations in the entire human body [8,9]. In some individuals, cancer driver somatic mutations were found in virtually all tissues, although none of them had been diagnosed with cancer. The most interesting recent finding is the presence of somatic *PTEN*, *KMT2D*, and *ARID1A* mutations in healthy liver cells [10]. Hepatocytes showing these well-known cancer driver mutations exhibited conspicuously increased fitness, faster expansion, and regeneration under stress or other injury as compared to their counterparts without mutation.

The study on liver cells showing high fitness and regenerative capacity despite their cancer driving mutation justifies the positive impact of somatic mutations on genomic stability rather than tumor promotion. There is a plausible explanation; the concentration of genome driver somatic mutations in tumors may not be pro-oncogenic stimuli, but may rather be DNA stabilizer actions via genomic plasticity. Somatic mutations in clinically cancer-free patients may derive from the earlier occurrence of accidental genomic instability or subclinical cancer in an organ, which were repaired or eliminated via activated mutations.

Molecular cancer therapies targeting the altered DNA damage response pathways lead to continuous failures. This problem evokes the idea that some modern cancer therapies might cause more harm than benefit, as we do not exactly understand the molecular events in the background of diseases [11]. The analysis of therapeutic failures urges a complete turn in our anti-cancer strategy rather than farther developing and improving the families of moderately effective or even genotoxic drugs.

The aim of this study is to justify that tumor cells are not enemies to be killed, butrather that they are ill human cells which have the remnants of the same regulatory pathways like patients’ healthy cells [12]. Understanding the real character and behavior of human tumors at the molecular level suggests that we should learn by watching the genome repairing methods of tumors instead of provoking additional genomic damages.

## 2. Endocrine Disruptor Synthetic Estrogens Increase the Risk for Certain Cancers and Cardiovascular Complications

In the early 1940s, synthetic estrogens were developed for medical purposes; for the treatment of miscarriage and menopausal complaints and later, for oral contraception. Diethylstilbestrol (DES) was a non-steroidal hormone; ethinylestradiol (EE) was a steroidal product; while conjugated equine estrogens (CEEs) were extracted from biological samples [13].

Increased breast cancer risk in DES-treated patients mistakenly suggested that synthetic estrogens activate the same subcellular pathways that a high endogenous estradiol level does, leading to alterations in all cellular functions including interactions with DNA [14]. In reality, malformations and increased breast cancer risk induced by prenatal exposure to DES may be attributed to the deregulation of estrogen signaling pathways. In animal experiments, DES and EE treatment provoked histone modification and further genomic damages via ER deregulation, justifying their endocrine disruptor character [15].

The development of synthetic estrogens, including both DES and EE, may be regarded as a pharmaceutical mistake as they are endocrine disruptors. Endocrine disruptors exhibit a special toxicological mechanism; higher doses induce more genomic damages as compared to lower doses; however, there are no safety low levels of these chemicals [16]. Low doses of synthetic estrogens exert an inhibitory effect on the ligand independent, ancient AF1 domain of ERs, while inducing compensatory estrogen-like activation on the ligand-dependent AF2 domain. Conversely, high doses of synthetic estrogens provoke a serious imbalance between the liganded and unliganded activation of ERs, resulting in uncompensated damages in the whole genomic machinery [17].

### 2.1. Controversial Correlations between Menopausal Hormone Therapy (MHT) and Women’s Health

For menopausal hormone therapy (MHT), both synthetic EE and CEE extracted from biological samples were prescribed [13]. From the 1940s, MHT became widely used among postmenopausal women for the treatment of menopausal symptoms and for the prevention of chronic illnesses, such as cardiovascular and thromboembolic complications and osteoporosis. In menopausal women, both natural and synthetic estrogens were applied alone or in combination with synthetic progestins as exogenous hormone therapies. Among HRT-using women, ambiguous clinical results were experienced; either increased or decreased risks for arterial and venous thromboembolism and for breast cancer was experienced. The guidance from the Food and Drug Administration (FDA) established that the benefits of MHT use surpass their risks [18]. Nevertheless, no comparative information was available on the efficacy and toxicity of synthetic versus natural hormone products.

In the early 2000s, two great Women’s Health Initiative (WHI) studies reported quite controversial results in women who underwent MHT. In 2002, increased risks for breast cancer, thromboembolism, and cardiovascular diseases were reported in menopausal women treated with conjugated equine estrogen (CEE) plus medroxyprogesterone acetate (MPA) [19]. Conversely, in 2004, another great WHI study reported on a striking reduction of breast cancer risk in women treated with CEE (Premarin, Pfizer) alone [20]. In the latter study, the protective effect of Premarin, with its natural origin, may be explained by the omission of the highly toxic progestin, MPA [21].

In 2019, a great meta-analysis study reported worldwide epidemiological evidence of the breast cancer-inducing capacity of MHT independent of the used hormone formuli and the timing of treatment [22]. All MHT studies reporting the breast cancer preventive effect of Premarin alone were omitted from this analysis. The concept of “estrogen-induced cancer” was both the starting point and the goal of investigation, creating a circular reasoning.

In 2020, the earlier WHI study was repeated on the surviving women eighteen years following the MHT, and the results reflected the long-lasting breast cancer preventive effect of Premarin. Both morbidity and breast cancer-associated mortality were significantly decreased among estrogen treated women [23]. These results justified the long term genome stabilizer power of natural estrogen treatment without synthetic progestin use [17].

In 2021, Premarin treatment of women with ER positive, PR negative breast cancers (N = 10,739) resulted in a significant reduction in tumors and breast cancer-related deaths. The authors established that here is the time for change in their breast cancer risk reduction strategies in clinical practice [24].

An analysis of the results of MHT studies using different hormone schedules justified that horse urine-derived Premarin without synthetic progestin is a highly beneficial formula against breast cancer, coronary heart disease, thromboembolism, and bone loss [21]. Although only synthetic hormones may be blamed for increased breast cancer risk and further complications in MHT-using women, the “estrogen-induced cancer” remained evidence-based fact.

### 2.2. Oral Contraceptives Are Endocrine Disruptors Inducing either Increased or Decreased Cancer Risk in Different Organs

Oral contraceptives (OCs) comprising synthetic EE were developed in the 1960s. OCs may induce serious toxic side effects, such as venous thromboembolism, stroke, and cardiovascular diseases [13]. OC use induced the deregulation of ER signaling and led to an increased risk for insulin resistance and metabolic diseases [25].

Wide spread use of OC use among premenopausal women caused highly ambiguous correlations with cancer risk at different sites. Among OC user women, a slightly increased risk for overall breast cancer was observed [26], while strongly increased risks for ER/PR negative and triple-negative breast cancer (TNBC) were registered [27,28]. Conversely, OC use significantly reduced the risk of endometrial [29], ovarian [30], and colon cancer [31]. The controversial correlations between OC use and reduced or enhanced cancer risk at different sites strongly justified that ethinylestradiol is an endocrine disruptor compound rather than a bioidentical estrogen [17].

In *BRCA* gene mutation carriers, long term OC use significantly increases the risk for overall breast cancer as compared to non-carriers [32]. Long term OC use in *BRCA* mutation carriers may exert an additional inhibition on the non-liganded ER activation aggravating mutation associated weakness of ERs. Conversely, in women, with *BRCA1/2* gene mutations, the risk for ovarian cancer is strongly reduced by OC use [33] via exerting an advantageous estrogen-like effect by the indirect activation of the AF2 domain [17].

Despite the known metabolic, thrombotic, and carcinogenic complications of OCs, they are widely used in medical practice. Clinicians do not believe, or do not want to believe, in the endocrine-disrupting nature of OCs. In addition, OC use strengthened the misbelief that endogenous estrogens in higher concentrations may induce increased breast cancer risk.

## 3. In *BRCA* Gene Mutation Carriers, the Defect of Liganded ER Activation Is the Initiator of DNA Damage and Cancer Development

Patients with the germline *BRCA* gene mutation are pathological models for genomic instability and have an increased predisposition for breast and ovarian cancer development. The first breast cancer gene (*BRCA1*) was identified in 1994, showing close correlation with breast cancer development when becoming mutated [34], while the second breast cancer gene (*BRCA2*) was announced in 1995 [35]. *BRCA1* and *BRCA2* genes may be regarded as safeguards of the genome. Their BRCA protein products control DNA replication, transcriptional processes, DNA recombination, and the repair of DNA damages [36].

Although functional BRCA proteins have crucial role in the health of all cell types in both men and women, germline *BRCA* gene mutations are preferentially associated with tumor development in female breasts and ovaries [37,38].

The tissue specificity of *BRCA1* mutation-associated tumors suggested a potential relationship between *BRCA1*-loss and excessive estrogen signaling in breast cancer development. However, *BRCA1* mutation-linked tumors are typically ER-alpha negative, poorly differentiated, and show rapid growth and poor prognosis [39]. Receptor expression profiling of *BRCA1* mutant tumors showed that their vast majority proved to be ER-alpha negative and ER/PR/HER2 negative, nominated as triple negative breast cancer (TNBC) [40]. In addition, the development of ER-alpha negative breast cancer has been reported to be a predictor of *BRCA1* mutation status in patients [41]. In sporadic ER-alpha negative breast cancers, reduced BRCA1 protein expression and a decreased level of ER-alpha mRNA were parallel observed, while estrogen treatment increased BRCA1/2 mRNA levels [42]. These results suggest that *BRCA* gene mutation deteriorates the regulatory interplay with ERs, leading to decreased ER expression and consequential decreased estrogen signaling [43].

Since the regulation of healthy female breast requires a strict balance between liganded and unliganded ER activation, the weakness in ER expression and estrogen activation results in a preferential susceptibility to genomic damage in the breasts of *BRCA* mutation carrier women [43]. In diabetes and obesity, weak estrogen signaling-associated defects in the hormonal and metabolic equilibrium are directly associated with an increased TNBC risk.

Molecular studies on the interactions between BRCA1 protein and ER alpha yielded highly controversial results supporting either the upregulating or downregulating effect of BRCA1 on ER alpha transactivation.

Wild type *BRCA1* gene was demonstrated to inhibit ER alpha transcriptional activity under the control of its estrogen responsive elements [44]. *BRCA1* could suppress the expression of near all estrogen-regulated genes [45]. In addition, *BRCA1* was able to inhibit p300 mediated ER acetylation, which is essential for the transactivation of ERs [46]. In contrast, it was reported that *BRCA1* may induce an increased transcriptional activity of ER alpha by the upregulation of p300 expression, a co-activator of ER alpha [47]. Similarly, BRCA1 ensured co-activator Cyclin D binding to ER alpha so as to facilitate the transcriptional activity [48].

These controversial findings reflect the complexity of regulatory processes, including both the activation and repression of ERs. In conclusion, estrogen-liganded ER alpha may choose momentarily appropriate cofactors, promoter regions, and transcriptional pathways in harmony with optimal BRCA1 expression and activation [49].

In genome stabilization, BRCA and ER proteins are in mutual interaction by direct binding regulating each other’s activation [50]. The amino-terminus of BRCA1 increases the activation of ER alpha, while the carboxyl-terminus of BRCA1 may function as a transcriptional repressor on the ER alpha protein. ER alpha and BRCA1 are crucial components of the regulatory circuit of DNA stabilization as well [49]. Defective expression or activation of either BRCA1 or ER alpha protein disturbs their interaction, endangering both estrogen signaling and genomic stability.

In women with the *BRCA* gene mutation, anovulatory infertility frequently occurs [51], reflecting the defects of the liganded estrogen signal. In addition, early menopause associated with ovarian failure is also a characteristic finding in *BRCA* mutation carriers [52]. In 85% of *BRCA1* mutation carriers, loss of functional BRCA1 protein correlated with elevated aromatase levels and increased estrogen synthesis [53] suggesting compensatory actions against decreased ER expression.

In *BRCA* mutation carrier breast cells, decreased BRCA1 protein synthesis is associated with the down-regulation of ER alpha mRNA expression and low ER alpha expression [54]. In *BRCA* gene mutation carrier tumor cells, a consequently decreased liganded activation of ERs was observed [44]. In *BRCA* gene mutation carrier breast cancer cells, a decreased expression of ER alpha was experienced [55].

The defect of liganded ER activation in *BRCA* mutation carriers is a crucial finding, as it explains the increased inclination for cancers, the ER negativity of developing tumors, and the ovulatory disorders of female patients.

### Both Healthy Cells and Tumor Cells with BRCA Mutation Show Compensatory Molecular Changes, Improving Genomic Stability

In BRCA mutation carriers, the defect in estrogen signaling endangers the genome stability in healthy cells, and means a risk for further genomic deregulation in tumor cells. In healthy cells with BRCA mutation, a compensatory upregulation of estrogen signaling may preserve genomic stability, while in BRCA mutation carrier tumor cells, increased estrogen signaling may protect from further genomic damage and increasing proliferative activity. Tumor cells possess the remnants of the same genome stabilizer pathways like healthy cells have. In the emergency situation of a weakening estrogen signal, tumor cells may show various activating mutations, increasing both liganded and unliganded ER activation [56].

Healthy cells: In mammary epithelial cells, the loss of the *BRCA1* gene leads to increased epidermal growth factor receptor expression [57], which means an unliganded activation of ERs instead of a pro-oncogenic impact. In *BRCA1* mutation carrier women, BRCA1 protein activity confers the selection of an appropriate *CYP19* aromatase promoter region for the compensatory intensifying of estrogen synthesis [58]. In mammary fibrous adipose cells, the downregulation of the *BRCA1* gene increased the specific activation of the PII promoter on *Cyp19* aromatase gene, leading to increased estrogen synthesis. The mutation of the *BRCA1* gene may be counteracted by the unliganded activation of ERs via the upregulation of growth factor receptors and P13K/Akt pathways interacting with BRCA1 protein [59].

Tumor cells: In *BRCA1*-deficient human ovarian cancer cells, ER alpha exhibited increased ligand independent transcriptional activity that was not observed in *BRCA1* proficient cells [60]. Authors suggested that the loss of *BRCA1* increased unliganded ER activation increasing cancer risk; however, it was a compensatory activation attributed to the defective liganded activation.

In the tumor cell line with *BRCA* mutation, increased estrogen signaling was observed via enhanced activation of p300, a transcriptional coactivator of ERs [47]. In familiar breast cancers with *BRCA* mutation, a further transcriptional activator of ERs—Cyclin D1—was highly accumulated [61]. Nuclear factor kappaB (NF-κB), an important ER coactivator, was persistently activated in a subset of *BRCA1*-deficient mammary luminal progenitor cells [62].

In *BRCA1/2* gene mutation carriers, the most frequently co-mutated gene was *TP53* (38.1%). Patients with both *BRCA1/2* and *TP53* gene mutations were more likely to have hormone receptor negative cancers, high Ki-67 values, and increased genetic mutations, especially of hormone receptor-related genes. Survival benefits were observed in the *BRCA2* mutation carrier patients with *TP53* co-mutation, compared to those with *TP53* wild types [63]. This valuable observation supports the increased genome stabilizer impact of mutated *TP53,* providing compensatory genome stabilization in tumors with *BRCA2* gene mutation.

In sporadic breast cancer cells, the wild *BRCA* gene is capable of increasing the expression of the coding gene of ER alpha—*ESR1*—mediated by the activator Oct-1 [55]. Moreover, *BRCA* could transcriptionally increase the expression of ER alpha mRNA.

Studies on *BRCA* mutation carriers teach us crucial new aspects for cancer research: 1. Genomic instability is linked to the weakness of liganded ER activation rather than excessive estrogen signaling; 2. *BRCA* gene mutation carrier healthy cells are working on the improvement of endangered DNA, via the upregulation of both liganded and unliganded ER activation; 3. In *BRCA* mutant tumor cells, the upregulation of estrogen synthesis and unliganded ER activation are efforts to protect DNA from further damage; 4. Both healthy and tumor cells with *BRCA* gene mutation exhibit gene amplification and activate gene mutations so as to increase estrogen synthesis and improve ER activation; 5. In *BRCA* mutation carriers, the whole body works on genome stabilization via increased ovarian and peripheral estrogen synthesis.

## 4. Estrogens Are the Principal Regulators of Genomic Machinery in Mammalian Cells

At the cellular level, estrogen-activated ERs (ER alpha and ER beta) are the hubs of genomic machinery, orchestrating all cellular functions affecting both somatic and reproductive health [64]. Molecular factors of all cellular processes are working in regulatory circuits. They receive the regulatory commands from estrogen-activated ERs directly or indirectly and, at the same time, send their signals back to the ERs closing the circuit.

ER-alpha-regulated DNA stabilizer circuit. ER-alphas activated by the estrogen hormone are the initiators and drivers of the regulatory circuit of DNA stabilization. ERs, genome safeguarding proteins, such as BRCA1, and estrogen synthesizing aromatase enzyme (A450) create a triangular partnership. The appropriate expression of ER-alpha, BRCA1 protein, and aromatase enzyme is harmonized by firm interplay among ESR1, BRCA1, and CYP19 genes and their transcriptional activity in the promoter regions [49]. The upregulation of estrogen signaling ensures DNA stability in all phases of cell proliferation.

Liganded ER-alpha as a transcriptional factor induces *ESR1* gene expression, driving protein coding ER-alpha-mRNA and ER-alpha protein expression. Liganded ER-alphas are capable of occupying the *BRCA1* gene promoter region as well, facilitating the expression of BRCA1 mRNA transcripts and increased BRCA1 protein synthesis [37].

The BRCA1 protein, as a transcriptional factor, drives the expression of the *BRCA1* gene and amplifies BRCA1 protein expression. The BRCA1 protein activates *ESR1* gene expression and increases ER-alpha protein synthesis [55]. Moreover, the BRCA1 protein is capable of occupying the promoter region of the *CYP19A* gene, conferring the augmented expression of the aromatase enzyme. The BRCA1 protein ensures safety equilibrium between the ER-alpha protein and aromatase enzyme expression [56]. Abundant BRCA1 proteins may induce epigenetic modification and activate mutations on *ESR1, BRCA1*, and *CYP19* aromatase genes via increasing the appropriate lncRNA expression and resulting in increased production of the three regulatory proteins: ER, BRCA1, and aromatase [56]. In addition, abundant BRCA1 proteins are capable of increasing the transcriptional activity of ER-alpha mediated by either Cyclin D1 [48] or p300 coactivator protein [47]. Increased *BRCA1* activity confers a decreased unliganded activation of ERs [60], while increasing liganded ER activation and strengthening DNA stability [17]. Some lncRNA transcripts of *BRCA1* may induce transcription on the *CYP19* aromatase promoter, facilitating A450 aromatase enzyme expression and estrogen concentration [58]. A high estrogen concentration helps in the binding and activation of abundant ER-alphas, further stimulating the DNA stabilizer circuit [49].

The process of estrogen-induced genome stabilization through the ER-BRCA-aromatase circuit may take many hours as protein synthesis is a time consuming procedure. In emergency situations, 17beta-estradiol can rapidly enhance aromatase enzyme activity and estrogen synthesis in both healthy and tumor cells. The non-receptor tyrosine kinase c-Src shows direct involvement in E2 stimulated quick aromatase activation via a short nongenomic autocrine loop [65].

ER-alpha and BRCA1 proteins can directly bind with each other as transcriptional factors. Certain binding sites facilitate upregulative processes, while others may quench each other’s transcriptional activity [50]. Mutagenic defects or the decreased expression of ER-alpha may dangerously repress the expression of BRCA1 mRNA transcripts and BRCA1-protein synthesis; endangering DNA-safeguarding [42]. Similarly, decreased synthesis or mutagenic alteration of BRCA1-protein results in the downregulation of the expression of the ER-alpha mRNA and ER-alpha protein [54]. If either the ER-alpha or BRCA1 protein function suffers damage, the result will be genomic instability and increased cancer risk [49].

ER-alpha-regulated circuit of cell proliferation. The principal regulator of cell proliferation is the ligand-activated ER-alpha in strong interactions with membrane-bound tyrosine kinase growth factor receptors; insulin-like growth factor receptor 1 (IGF-1R) and epidermal growth factor receptor (EGFR) [12]. The equilibrium between liganded and unliganded ER-alpha activation provides an accurate control over DNA replication in both high and low phases of cell proliferation. The interplay between ER and GFR receptor families is the prerequisite of the regulation of cell growth and proliferation and it may be more or less preserved even in malignant tumors [17].

IGF-1R shows a bidirectional signaling pathway with ligand-activated ERs [66]. IGF-I expression is influenced by both insulin and growth hormone (GH) stimulating the IGF-I synthesis in the liver [67]. IGF-1 binding to its receptor, IGF-1R may upregulate two chief signaling pathways: the phosphatidyloinositol 3-kinase (PI3K-AKT) and the Ras-mitogen-activated protein kinase (MAPK) pathways. These kinase cascades drive the unliganded transcriptional activity of ER-alpha by the phosphorylation of serine residues [68].

ERs are driving many protein components in the insulin-IGF-1 system, such as the IGF-1R and insulin receptor substrate 1 (IRS-1) [69]. ER-alpha is capable of binding and phosphorylating IGF-1R and taking care of its signaling pathways. In IGF-1 KO mice, estradiol-activated uterine growth is missing [70]. Conversely, in vivo IGF-1 activation of uterine cell proliferation is strongly dependent on ER-alpha activation [71].

Estrogen stimulates the EGF synthesis in uterine epithelial cells through ER activation, resulting in a proliferative effect [72]. In estrogen-free milieu, EGFR signaling may be activated through unliganded ER activation [73]. In turn, in the uterus of ER-alpha KO mice, EGF could not induce DNA synthesis and transcriptional activity [74]. In ovariectomized mice, estradiol treatment resulted in a rapid increase in uterine EGFR mRNA and protein expression and increased the binding sites on EGF through ER activation [75].

In the nucleus, the EGFR signal induces phosphorylation and activation on ER-alpha at serine 118 location conferred by the growth factor receptor-activated MAPK pathway [76,77]. Phosphorylation at serine 118 increases the ER-associated transactivation of several genes that are activated by EGFR. The growth factor receptor signal is capable of increasing the transcriptional activity of nuclear ERs through the phosphorylation of their coactivator proteins, such as steroid receptor coactivator 1, p300 protein, and cyclin D1 [78,79].

In the cytoplasm, estrogen-activated ERs induce EGFR activation and EGFR conferred upregulation of the PI3K signaling pathway [80]. In endothelial cells, estrogen treatment induced PI3K activation resulted in the rapid upregulation of 250 estrogen-regulated genes within 40 min [81]. The ER/EGFR interplay at the membrane promotes the activation of numerous signaling pathways that further increases the wide-ranging transcriptional activity of ERs [66].

In human breast cancer, an inverse correlation may be observed between ER and EGFR expression [82,83]. In breast cancer cell lines responsive to tamoxifen, a counteractive increased expression of ERs may be experienced, improving estrogen signaling. In tumors non-responsive to tamoxifen, an additional increased expression of growth factor receptors may be experienced [84], conferring the unliganded activation of ERs. Abundant GFRs highly increase ER activation via unliganded pathway; however, they cannot compensate the tamoxifen blockade of AF2 domain [17].

ER-alpha-regulated fuel supply circuit. Liganded ER-alpha drives a regulatory circuit to maintain glucose homeostasis and to stimulate all the phases of cellular glucose uptake providing fuel for all cellular functions [49]. Defects in the estrogen signal results in serious alterations in cellular glucose uptake designated as insulin resistance and leads to serious chronic diseases including cancer [85]. In conclusion, insulin resistance is the linkage between a weak estrogen signal and increased cancer risk.

Estrogen-regulated genes activate insulin synthesis and secretion, as well as the expression and activation of insulin receptor [86]. When insulin binds to its receptor, autophosphorylations of multiple tyrosines induce the activation of insulin signal transduction [87]. Liganded ERs upregulate the expression and functional activity of intracellular glucose transporter-4 (GLUT4), promoting insulin-assisted glucose uptake [88]. Liganded ER-alpha drives the insulin receptor substrate 1 (IRS1) conferred activation of PI3K/mTOR signaling pathway which ensures the hormone free activation of nuclear ERs [89].

Estrogen signal activates glucose uptake even in cancer cells supplying energy for the self-directed improvement of DNA stability. In the MCF-7 breast cancer cell line, estradiol enhances the expression of the insulin receptor substrate-1 (IRS-1), activating insulin signaling [90]. In ZR-75-1 breast cancer cells, estrogen/progesterone treatment increased glucose transporter 1 (GLUT1) expression [91]. In MCF-7 cell lines, estradiol treatment activated ERs via the PI3K/Akt signaling pathway and, at the same time, increased the translocation of glucose transporter 4 (GLUT4) vesicles to the plasma membrane [92]. A defective or blocked estrogen signal results in the failure of glucose uptake even in cancer cells, declining the activity of genome stabilizer pathways.

## 5. Estrogens Are Master Regulators of Metabolism and Energy Homeostasis via Orchestrating Adipose Tissue Functions

Adipose tissue, deposited all over the body, provides energy and epigenetic regulatory commands for all tissues and organs via its estrogen-activated ER network. In healthy adipose tissue, estrogen signaling regulates the glucose homeostasis and the balance of lipolysis/lipogenesis [93,94]. In adipose tissue, damaged estrogen signaling leads to defects in all regulatory functions, and serious diseases may develop in the fat-regulated visceral organs, cardiovascular structures, and hemopoietic bone marrow [95].

The subcutaneously located adipose tissue provides energy and estrogen regulation for the skin and the skeletal muscles. Centrally positioned fatty tissue within the trunk and abdomen closely surrounds the visceral organs and cardiovascular structures [96]. Visceral fat is largely located in the omental and mesenteric adipose tissue in the vicinity of stomach, intestines, liver and pancreas. Kidneys, and the attached adrenal glands, are embedded into abundant fatty tissue capsule. Adipose tissue deposition within the visceral pericardium surrounds the myocardium and coronary arteries providing estrogen signaling and energy for the moving heart. Perivascular adipose tissue nurses most blood vessels, with the exception of the pulmonary and cerebral arteries [97]. A further depot of adipose tissue is gonadal fat (GAT) surrounding the ovaries and testes having specific regulatory functions [98].

Female breasts enjoy an exceptional nursing level as mammary lobules are intimately intermingled with the estrogen and ER rich fatty tissue pad [99]. This close connection between the adipocytes and mammary cells is associated with the extreme demand of breasts for strict regulatory control and abundant energy supply. The high claim of breasts for regulatory commands may explain their unique vulnerability to estrogen loss or defects in ER activation.

The third largest fat depot is the bone marrow fat, following subcutaneous and visceral fatty tissue. Adipocytes are active components of the bone marrow microenvironment, regulating hemopoietic and immune cell proliferation and function via their estrogen signal and secretome [100].

Interestingly, the central nervous system does not enjoy the estrogen driven adipose tissue safeguard, while the brain shows an extreme claim for estrogen regulation. Recently, microbial sequences were found in healthy human brain samples [101] suggesting that they may provide important support for cerebral functions. Microbiom in the gut has great role in increasing unbound, free estrogen levels via their β-glucuronidase activity [102,103]. It is a plausible possibility that gut microbiom colonized in the brain increases the level of accessible free estrogen.

Adipose tissue is an essential source of estrogen production in extragonadal sites in both women and men [104]. The functional activity of adipose tissue is regulated by circulating and locally synthesized estrogens. In the fatty tissue, estrogens are acting in an autocrine manner, while in the adjacent organs; they increase ER activation in a paracrine manner [105]. Estrogens are the chief regulators of the health of adipose tissue through metabolic and epigenetic pathways [106]. Estrogen exerts its special effects on estrogen responsive adipocytes by estrogen receptors (E-alpha, ER-beta and GPR30) [107].

In the gonads, the essential precursors of estrogen synthesis are C19 steroids, while extragonadal sites are unable to synthesize estrogens directly from these factors. With ageing, increasing estrogen synthesis in peripheral tissues requires a precursor supply from external sources, for example, dehydroepiandrosterone (DHEA) intake is important [108].

The remarkable volume of ubiquitous fatty tissue and its noteworthy estrogen synthesis justify that fat cells have crucial roles in safeguarding and regulating the signaling network of neighboring tissues, organs, and the whole body.

### Secretory Activities of Visceral Adipose Tissue in Healthy Lean and Obese Cases

Abdominal fatty tissue has crucial secretory functions [109]. Estrogen-regulated genes orchestrate adipokine, cytokine, and growth factor secretion, which are important signaling molecules and their estrogen-regulated activation controls the health of the whole body.

Sexual steroids: In adipose tissue, estrogens are the crucial sexual steroids. Appropriate estrogen signaling controls the expression of numerous genes and the coordinated synthesis of signaling molecules [106].

Adipokines: Leptin controls the equilibrium of energy in the hypothalamus, conferring anorexinogenic and lipolytic signals. Estrogen treatment results in the increased expression of leptin receptors in various cells, sensitizing them to leptin [110]. In aromatase knock out (ARKO) mice with estrogen loss, visceral fat deposition develops and leptin levels are highly elevated [111]. Adiponectin signaling protects against insulin resistance by quenching various inflammatory reactions and improving endothelial functions. In adult mice, oophorectomy increases adiponectin levels, while it may be reduced by estradiol substitution [112]. Obesity increases the level of resistin, which may be a compensatory response. In subcutaneous fat cells, an estradiol benzoate treatment decreases resistin levels [113].

Proinflammatory cytokines and low-grade inflammation: Proinflammatory cytokines are regulatory proteins which have a great role in the maintenance of genomic and metabolic stability. In obese fatty tissue, low-grade inflammatory reactions and abundantly expressed cytokines are counteractions to genomic deregulation via increasing estrogen synthesis [114]. The insulin resistance of obese estrogen deficient adipose tissue leads to further regulatory disorders in the adjacent organs, resulting in serious co-morbidities, such as fatty degeneration and malignancies [115,116].

In the low-grade inflammation of obese adipose tissue, increased levels of inflammatory cytokines and immune cell infiltration comprising macrophages and T cells may be found [117]. Proinflammatory cytokines, including tumor necrosis factor alpha (TNF-*α*) and interleukin-6 (IL-6) generate an increased expression and activation of the aromatase enzyme, resulting in increased estrogen synthesis [118]. Proinflammatory cytokines have beneficial effects against obesity and obesity-related metabolic disorders via increasing the aromatase activity and estrogen synthesis. Estrogen treatment of obese ovariectomized mice decreased the expression of inflammatory cytokines, including TNFα and upregulated estrogen signaling, which improved the insulin sensitivity in both adipose tissue and liver [119].

Insulin-IGF system. The insulin-like growth factor (IGF) system has a great role in the regulation and control of growth and differentiation. The receptors of insulin and insulin-like growth factors work as ligand-specific modulators, regulating various genes on similar pathway [120]. In the early stage of insulin resistance, an increased IGF-1 level confers increased insulin synthesis, leading to compensatory hyperinsulinemia.

Harmonized crosstalk and interaction among signaling pathways of ERs and growth factor receptors (IGF-1R, EGFR, VGFR) are identified in both health and disease [121,122]. In health, growth factor-activated ERs may either facilitate or silence cell growth and proliferation. In tumors with regulatory defects, abundant growth factor receptors activate ERs via unliganded pathway so as to initiate DNA stabilization and apoptotic death rather than providing excessive proliferative stimulus.

In adipocytes, estrogens control the synthesis of insulin-like growth factor 1 (IGF-1) and the expression of its receptor (IGF-1R). In turn, the upregulation of IGF-1 synthesis and its receptor expression increases the unliganded activation of ERs via the AKT and MAPK regulatory pathways [123]. In an estrogen deficient milieu, increased IGF-1 receptor signaling stimulates the unliganded activation of ERs, which may momentarily ensure the genome wide expression of estrogen-regulated genes [64]. In conclusion, in insulin resistance and obesity, the increased activation and expression of IGF-1 receptors do not exert pro-oncogenic effects, but rather facilitate unliganded ER activation.

Interaction between adipocytes and immune cells. Adipocytes are in signaling crosstalk with immune cells in both healthy and obese adipose tissue. In lean adipose tissue, IL-4 secreted by eosinophil granulocytes and regulatory T (Treg) cells activate M2 type macrophages, which express arginase and anti-inflammatory cytokines such as IL-10. In contrast, in obese adipose tissue, a high number of M1 type macrophages and increased secretion of pro-inflammatory cytokines, such as TNFα and IL-6, are coupled with a decrease in anti-inflammatory immune cells [117]. In animal experiments, estrogen is capable of improving metabolic disorders and, at the same time, exerts anti-inflammatory effects. In female mice, estrogen protects from adipocyte hypertrophy, obesity, and prevents adipose tissue oxidative stress and inflammation [124].

In obesity, the upregulation of estrogen signaling restores insulin sensitivity, reduces lipid deposition, decreases pro-inflammatory cytokine synthesis and quenches inflammatory infiltration. Estrogen treatment provides quite new ways for the prevention and cure of obesity and obesity-related complications.

## 6. The Tumor Cell Itself Is the Frontline of Anticancer Combat

According to global medical concepts, tumor cells are enemies to be killed as they presumably fight for their survival, similar to how pathogenic bacteria fight against antibiotics. Seemingly, tumor cells express cancer driver genes via somatic mutation, and their altered protein products defeat both the immune defense of body and the therapeutic effect of pharmaceutical agents.

In reality, the recognition of DNA damage means an emergency state even for tumor cells. The upregulation of estrogen signaling via the liganded and/or unliganded pathway is the appropriate means for the restoration of DNA stability. However, in tumors, the possibility for DNA repair is questionable, attributed to the genomic damage. The more differentiated a tumor, the stronger its capacity for the compensatory upregulation of estrogen signaling, coupled with DNA restorative efforts [125].

The spontaneous healing of early breast tumors is a well-known finding justifying the capacity of initial cancers for self-directed remission. A systematic review and meta-analysis study evaluated a high prevalence of incidental breast cancer and precursor lesions in autopsy studies on clinically tumor-free cases. The estimated mean prevalence of incidental cancer and precursor lesions were surprisingly high: 19.5% and 0.85% [126].

Breast cancer is regarded as a multifactorial and very heterogeneous disease that refers to the abnormal proliferation of the lobular and ductal epithelium of the breast, resulting in tumor formation [127]. The classifications of breast cancers follow the recommendations of the World Health Organization (WHO), which are regularly revised in accordance with the scientific progress [128].

The most important parameter for the classification of breast cancers is their molecular profile as it was described in 2000 [129]. The heterogeneity of breast cancers at a molecular level was revealed through the various expression of a panel of genes. Breast cancers were divided into four main groups: 1. Luminal A (60% of cases); 2. Luminal B (10% of cases); 3. The overexpression of human epidermal growth factor receptor2 (HER2) (20% of cases); and 4. Basal-like triple-negative breast cancers (TNBCs) (about 10% of breast cancers). Another subgroup has also been described as a normal breast-like subcategory which resembles the luminal A group but shows a worse prognosis.

In clinical practice, these tumor groups are identified by immunohistochemical markers, such as ER-alpha, progesterone (PR), and human epidermal growth factor receptor (HER2) expression [127]. In breast cancers, the overexpression of certain receptor families is mistakenly regarded as an aggressive survival technique and their targeted inhibition is the principle of current therapeutic measures. In reality, missing or decreased expression of certain receptors in tumor cells highlights the points of genomic defects requiring repair. Conversely, the overexpression of certain receptors and regulators, as well as the activating mutation of their genes indicate the efforts for self-directed genomic repair of tumors rather than developing survival techniques [12,56]. In reality, the loss of certain receptors indicates the genomic damage, while the overexpression of others represents the genome repairing effort.

Immunohistochemical markers of breast cancers show the alterations in their gene and receptor protein expression as compared to healthy breast epithelium. Molecular alterations reflecting the grade of DNA damage and the concomitant DNA repairing actions in different breast cancer subtypes are shown in Table 1.

Luminal type A cancers are the least aggressive tumors with the expression of ER alpha, and PR. Increased ER expression in breast tumors is traditionally regarded as a crucial inducer and promoter of tumor growth [127]. This concept derives from confusing the constellation with causation. Increased ER expression is not a causal factor for tumor growth, but rather it is an effort for improving estrogen signaling and DNA stabilization in an estrogen deficient milieu [43].

Estrogen receptor expression was shown to be parallel with DNA repair capacity in breast cancer cells [130]. This correlation justifies that the high ER expression of untreated tumors is the key to self-directed DNA repair, rather than a fuel for tumor growth. The strong belief in estrogen induced cancer does not allow consideration of opposite alternatives.

Luminal A breast cancer may exhibit a transiently good response in 50% of tumors to adjuvant endocrine therapy; however, near all patients previously showing good tumor responses later become non-responders [131]. Patients with early luminal ER-positive breast cancer are at a continuous risk of relapse even after more than 10 years of tamoxifen treatment [132]. These experiences underline that endocrine disruptor therapy is not appropriate method even for early ER-positive breast cancer care.

Luminal B tumors are more aggressive than luminal A types. They express lower ER alpha and lower PR expression or may be PR-negative, in correlation with the weakening estrogen signal [133]. Luminal B tumors are associated with an increased rate of p53 mutations and in certain B type tumors, HER2 may also be expressed [134]. Activating p53 mutations are not oncogenic changes, but rather they mean stronger DNA protection in tumors with weakening genome stability. In luminal B type tumors, the appearance of HER2 expression works on the compensatory unliganded activation of ERs [17].

After tamoxifen therapy, patients with ER-positive, PR-negative, and HER2-positive tumors exhibited higher rates of tumor recurrence and mortality as compared to those who did not receive the agent [135]. This observation suggests that in type B tumors, the weakening ER signal is further worsened by endocrine disruptor treatment. In contrast, Premarin treatment of ER-positive, PR-negative breast cancer cases resulted in a significant reduction in tumor size and improved patients’ survival [24].

HER2-enriched breast cancer is ER- and PR-negative and HER2-positive. HER-2-enriched cancers tend to grow faster than luminal cancers and can have a worse prognosis. ER- and PR-negativity in HER-2 enriched breast cancers reflects a loss of estrogen signaling and strong defects in all genomic processes. HER2 overexpression in hormone receptor negative tumors is mistakenly regarded as a trigger for tumor proliferation, similarly to all other growth factors [127]. In contrast, in the emergency situation of DNA damage, HER-2 overexpression is a compensatory effort for the unliganded activation of ERs occurring scarcely in this tumor type [17]. HER-2 protein-targeted therapies against HER-2-enriched tumors show similarly ambiguous results, like ER-inhibitor anti-estrogens against ER-positive tumors [12].

Triple-negative or basal-like breast cancer is ER-negative, progesterone receptor-negative, and HER-2-negative. Triple-negative breast cancer is more common in people with BRCA1 gene mutation, younger women, and black women. Triple-negative breast cancers are more aggressive than either luminal A or luminal B breast cancers and they are not responsive to endocrine therapy [127].

In triple negative breast cancers (TNBCs), the lack of ER, PR, and HER-2 receptors indicate the serious deregulation of the whole genomic machinery. These tumors are poorly differentiated and clinically show rapid growth and spread. In TNBC type tumors, there is no possibility for self-directed DNA repair as ERs seem to be absent or hidden and the regulatory pathways for both liganded and non-liganded ER activations are unnoticeable [43]. The increased risk for TNBC-type tumors in African American women may be attributed to their excessive pigmentation in a relatively light-deficient geographical region. Poor light exposure leads to metabolic and hormonal alterations, conferring an increased cancer risk [136].

The molecular classification of breast cancer types reflects the fact that in women, stronger estrogen signaling may suppress, while a defective estrogen signal liberates breast cancer initiation and growth [43]. In tumor cells, the higher the ER expression, the stronger is the apoptotic effect of therapeutic estrogen exposure. In contrast, endocrine disruptor therapies may achieve only transient tumor responses in appropriately ER-positive breast cancers. Poorly differentiated ER/PR-negative and TNBC-type tumors are refractory to anti-estrogen therapy, attributed to their serious genomic deregulation.

In conclusion, breast cancers are not multifaceted tumors with quite different etiology and pathogenesis. Consequently, they do not need quite different therapies depending on their receptor status. The levels of regulatory defects create a line of variously differentiated tumors between strongly ER-positive, highly differentiated, and poorly differentiated TNBC-type ones. In breast cancer therapy, natural estrogen is a risk-free available option for ER-positive tumors [24]. Against ER-negative and TNBC-type poorly differentiated tumors, Maloney’s mRNA technology would be a promising therapy to be introduced in the near future [125].

## 7. Peritumoral Microenvironment: The Second Line of the Antitumor Battle

In the early 2000s, the role of the tumor microenvironment emerged as being an important player in cancer development, tumor invasion, and metastatic spread [137]. Today, cancer is regarded as a complex disease built up from the neoplastic lump and its altered cellular and stromal microenvironment [138,139]. There is a strengthening belief that tumors insidiously influence all players in their microenvironment via dynamic intercellular communication. Tumors presumably ensure their invasive growth via escape from defensive immune reactions and anti-cancer treatment.

The supposed conspiration between tumors and their microenvironment is based on the belief that all signaling molecules and regulatory proteins are taken for pro-oncogenic factors when their expression is highly elevated in tumors and in the adjacent cellular infiltration [139,140,141]. In addition, when important regulatory genes, such as *ESR1*, are accumulated or mutated in tumors, they are regarded as pro-oncogenic alterations, rather than self-regulated efforts in the repair of genomic damages [142,143,144,145,146]. According to the reigning preconception, in tumor cells, the upregulation of estrogen signaling and its activator pathways are regarded as the keys to tumor growth.

In reality, in tumors, the upregulation of certain signaling pathways and activating mutations are not pro-oncogenic factors, but rather they are efforts for metabolic improvement and genomic stabilization [56]. Unfortunately, advanced tumors have weakened capacities for self-directed genomic repair and they ask for help via sending messages to their microenvironment. In turn, peritumoral-activated cells send signals and regulatory molecules, helping the tumor to achieve DNA repair and to commit apoptosis as a kamikaze action.

The re-evaluation of studies on the biochemical and genomic communication between tumors and activated microenvironmental cells revealed that all signal messages and transported exosomes aim for the upregulation of each other’s estrogen signaling and the improvement of all genomic functions. These activating processes serve the elimination of the tumor rather than helping its proliferation and invasion. In conclusion, the dynamic communication between the tumor and its microenvironment is a marvelous collaboration among molecular players fighting for the genomic repair and apoptosis of tumor by means of their genomic plasticity.

Cancer-associated fibroblasts (CAFs) are major components emerging in the tumor microenvironment. Their assembly and activation may be attributed to signals deriving from cancer cells [138]. CAFs are in continuous signal communication with cancer cells and all other cell types in the tumor microenvironment [139]. Distant intercellular communication occurs by spherical extracellular vesicles (EVs) comprising exosomes carrying different molecules, such as proteins, DNAs, non-coding RNAs, miRNAs, and mRNAs. Biochemical and genetic cross-talk between cancer cells and CAFs are important observations; however, the presumed cooperation for tumor invasion and metastatic spread is not justified, it is a biased labeling.

Activation of growth factor signaling cascades. In CAFs, the expression of growth factors, such as the insulin-like growth factor (IGF-1), fibroblast growth factor FGF-7, FGF-10, HGF, and TGF-beta 2 are regarded as pro-tumorigenic factors [147]. In reality, estrogen receptors and growth factor receptors are common regulators of crucial cellular functions including cell growth and apoptosis, as well as metabolic processes even in tumors [66].

Transforming the growth factor beta (TGF-beta) superfamily is the main inducer of CAF activation and in turn, CAFs secrete large amount of TGF-beta isoforms for improving tumor cell regulation [148]. Tumor cell-derived extracellular vesicles (EVs) may frequently contain growth factor TGF-beta, which is regarded as a typical mitogen factor of tumors [149]. Considering the ER-activating role of growth factors, tumors send them to CAFs for the activation of their estrogen signal. Tumor-derived EVs, containing certain miRNAs, contribute to the enhanced TGF-beta expression in CAFs through the phosphoinositide 3-kinase (PI3K)/protein kinase B (AKT)/mammalian target of rapamycin (mTOR) signaling pathway [150]. PI3K and AKT/mTOR pathways upregulate ER activation and improve glucose uptake, which are not pro-tumorigenic processes, but rather increase anti-tumor activity. Cancer cell-derived EVs, containing mRNA coding for CXCR-4 and IGF-1R, provoke CAFs for growth factor secretion in acute myeloid leukemia [151].

Cytokines secreted by CAFs, macrophages and immune cells are important regulators of inflammatory processes and immune reactions in the tumor microenvironment [152]. Estrogen signaling orchestrates the secretion of both pro-inflammatory and anti-inflammatory cytokines according to the momentary requirements. Pro-inflammatory cytokines stimulate aromatase activity, estrogen synthesis and ER expression in the estrogen responsive peritumoral cellular infiltration. When estrogen concentration reaches an appropriately high concentration, the accumulation of anti-inflammatory cytokines will quench the inflammatory reaction parallel with the decreasing estrogen level [114].

IL-1β accumulation in hyperplastic lesions activates CAF formation from fibroblasts via the NF-κB pathway [153], which is a coactivator of ERs, promoting genome stabilization. Proinflammatory cytokines, IL-6 and TNF-α, are capable of aromatase activation, leading to increased estrogen concentration and the upregulation of estrogen signaling [154]. In gastric cancer, tumors send miRNA containing vesicles to CAFs so as to induce inflammatory cytokine/chemokine secretion through the Janus kinase (JAK)/STAT and NF-κB signaling pathways [155]. In colorectal cancers, the constitutive mutation of KRAS increases the activation of EGFR kinase cascades PI3K-Akt and RAS-RAF-MAPK, whereas increases RAS-GEF signaling pathway, which is related to abundant cytokine production [156]. In Hodgkin lymphoma, CAFs exposed to tumor cell-derived EVs show increased proinflammatory cytokine secretion [157]. CAFs activated by tumor EVs, may in turn shed additional EVs that will transfer signaling and regulatory molecules to tumor cells.

Various tumors promote aromatase activity and estradiol synthesis in the peritumoral stroma via the promotion of proinflammatory cytokine secretion [158]. In breast cancers, aromatase is abundantly expressed in tumor cells, intratumoral fibrous cells, and neighboring adipocytes, justifying their collaboration in promotion of excessive estrogen synthesis [159]. These observations mistakenly support the role of increased estrogen concentration in tumor growth and invasion.

In contrast, a combined genetic and clinical investigation justified the anti-cancer capacity of increased local estrogen synthesis in tumors and their stroma. In a large prospective study, the examination of the surgical breast tumor samples revealed a significant correlation between a low aromatase level and an increased loco-regional recurrence rate of tumors [160]. This study suggests that missing estrogen synthesis in tumors is associated with worse prognosis in breast cancer cases.

Circulating estradiol may be systemic modulator of CAF secretome as CAFs express steroid receptors [161]. Estradiol regulates the expression of several microRNAs in CAFs deriving from breast cancer [162]. In gastric cancer, estrogens stimulate IL-6 secretion of CAFs, promoting the signal transducer and activator of transcription (STAT-3) expression [163]. The increased expression of STAT3 in CAFs secretome confers an effort for genome stabilization, as STAT3 is a transcription factor which has an important role in DNA replication.

Few studies evaluated growth factors and cytokines as positive regulators of the genome rather than pro-tumorigenic factors. TGF-beta was considered as a tumor suppressor factor due to its cytostatic effect on cancer cells [164]. IL-11 was known for its capacity to stimulate platelet production in cancer patients with thrombocytopenia [165].

Immune cells in the tumor microenvironment show intense interactions with tumor cells. The interaction between immune cells and other cell types are regulated by cell surface immune checkpoints [138].Mast cells are recruited near tumors during tumorgenesis and release a variety of cytokines and chemokines [166]. Cytokines and chemokines are crucial regulators of both genomic and immunologic processes and their accumulation is an anti-cancer effort. Natural killer cells (NK) are cytotoxic and secrete tumor necrosis factor so as to kill tumor cells [167].

Tumor-associated macrophages (TAMs) infiltrate the microenvironment of tumors and are mainly divided into two categories: classically activated macrophages (M1 type) and alternatively activated macrophages (M2 type). The activated M2 type macrophages are blamed for managing the immune escape of tumors. The abundance of TAM infiltration in tumors is mechanically linked with poor disease prognosis [168]. TAM activation and accumulation in tumors is not a pro-oncogenic feature, but rather their intensive cytokine secretion is helping aromatase activity and increasing estrogen concentration.

Myeloid-derived suppressor cells (MDSC) have apparently immunosuppressive effects; they may block immunotherapy and may play a role in tumor maintenance and progression [169]. MDSCs also accumulate in response to the chronic inflammation and lipid deposition in obesity and contribute to the more rapid progression of cancers in obese individuals. In reality, the accumulation of MDSCs is not a causal factor of rapid tumor progression and obesity associated inflammation, but rather it seems to be an intense immune defense against metabolic disorder associated tumors.

Tumor-infiltrating lymphocytes (TILs) are important participants of the tumor microenvironment [152]. Immune cell infiltrates may exhibit ambiguous properties, either promoting or inhibiting tumor progression depending on the features of the primary tumor [170]. CD4^+^ T cell polarization has been identified as a mediator of tumor immune surveillance. T helper 1 (Th1) cell functions are associated with tumor suppression and the upregulation of IFNγ and IL-12. T helper 2 (Th2) responses are reliant on IL-4 production and presumably exhibit tumor-promoting activity [171,172]. Murine and human studies reported that increased E2 concentration induces increased Th2 responses and upregulates IL-4 secretion [173,174].

A remarkable fact is that constellation of strong estrogen signal and increasing tumor growth does not justify causal correlation. A recent study reported increased immune cell infiltrate comprising Th1 T cells, B cells, and cytotoxic T lymphocytes (CTLs) in ER-negative breast tumors as compared to ER-positive cancers [175]. The correlation between ER-negative breast tumors and more intensive immune cell infiltration strongly suggests that poorly differentiated tumors with a loss of estrogen signaling need stronger immune support for their DNA repair than highly differentiated ER-positive ones.

Gene expression analysis in ER-positive breast cancer patients showed that blocking the liganded ER activation with aromatase inhibitor (letrozole) continuously increased the tumor infiltration with B cell and T helper lymphocyte subsets following treatment initiation [158]. This result justified that letrozole inhibition of estrogen signal in ER-positive tumors induced an emergency state, promptly recruiting strong immune cell infiltration.

In conclusion, tumors and their microenvironment are allies in the fight against worsening genomic defects and consequential tumor invasion. The more serious the genomic damage of a tumor, the denser is the peritumoral immune cell infiltration attributed to the emergency state. Invasive tumor spread, coupled with intensive peritumoral cellular infiltration, may be regarded as a common failure of tumor and peritumoral cells rather than the victory of presumably conspirator partners.

## 8. Molecular Changes in Tumors Responsive and Non-Responsive to Endocrine Therapy

The traditional belief of estrogen-induced breast cancer required the introduction of inhibitors of estrogen signaling for breast cancer care. The pharmaceutical industry developed two kinds of anti-estrogens for therapeutic purposes: a selective estrogen receptor modulator—tamoxifen—and an aromatase inhibitor (AI)—letrozole [176]. Since the early 1970s, anti-estrogens are commonly used compounds for breast cancer care as adjuvant therapy.

In breast cancer cases, anti-estrogen therapy caused many difficulties from the onset because of the development of so-called endocrine resistance in tumors. Results of anti-estrogen use could not surpass the “magic” 30% of tumor response rate, showing similar weaknesses to other endocrine therapies like oophorectomy or high dose synthetic estrogen [177]. About 70% of overall breast cancers could not respond to anti-estrogen therapy, showing stagnation or an even faster growth. Moreover, about half of the targeted ER-positive breast cancers exhibited primary resistance to anti-estrogen treatment [131]. Moreover, near all patients showing earlier good tumor responses to endocrine treatment later experienced secondary resistance, leading to metastatic disease and a fatal outcome [178].

In the past decades, great efforts were exerted for revealing the mechanism of presumed endocrine resistance of ER-positive breast cancers so as to predict responses to adjuvant endocrine therapy in patients. Researchers mistakenly supposed that both responsive and non-responsive tumor cells are aggressive enemies, developing various techniques in fighting for their survival [12].

### 8.1. Successful Fight of Anti-estrogen Responsive Tumors against the Endocrine Disruptor Treatment

**Figure 1:**
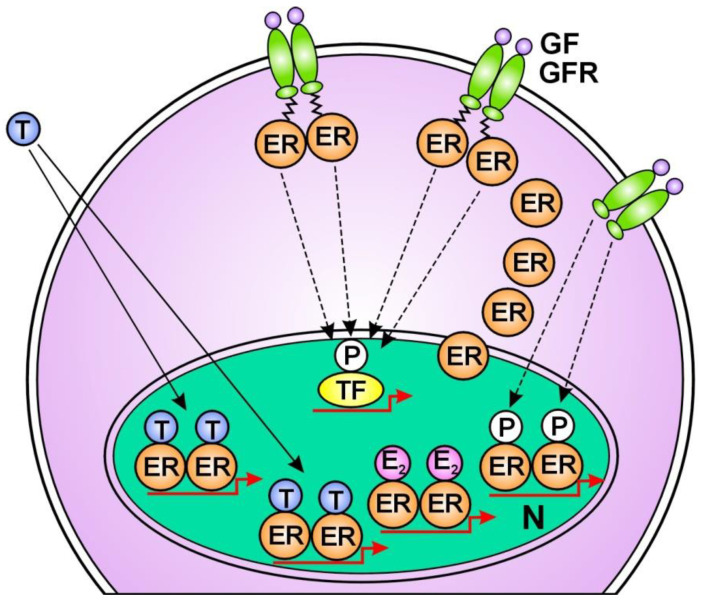
Rapid response to Tamoxifen (T) induced ER blockade in cancer cells.The rapid translocation of unbound estrogen receptors (ERs) out of the nucleus helps their interactions with membrane-associated growth factor receptors; GFRs (IGF1-R, EGFR). Cytoplasmic ERs activated by growth factor receptors initiate rapid transcriptional processes in the nucleus via transcriptional factors (TFs). Growth factor (GF)-activated GFRs may also induce unliganded activation on nuclear unbound ERs, driving their transcriptional activity. E: estrogen, P: phosphorylation, N: nucleus, Dotted arrow: activation, black solid arrow: inhibition, red arrow: schematic DNA segment.

**Figure 2:**
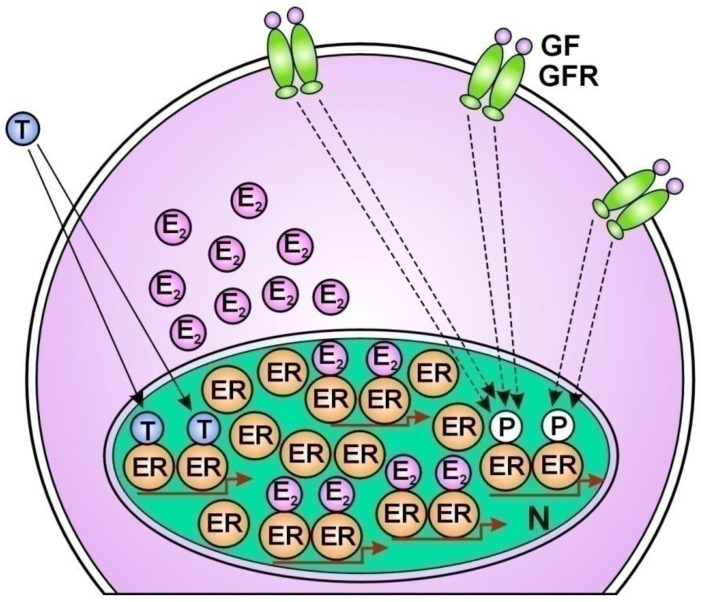
Molecular mechanism of tumor response in Tamoxifen (T) treated cancer cells. Increased estradiol (E_2_) concentration activates newly expressed abundant estrogen receptors (ERs) increasing the expression of estrogen-regulated genes. In the meantime, growth factors (GFs) activate growth factor receptors (GFRs) conferring unliganded activation for free nuclear ERs. The predominance of estradiol (E_2_) bound ERs over T bound ones leads to DNA repair, apoptotic death and clinical tumor response. P: phosphorylation, N: nucleus, Dotted arrow: activation, black solid arrow: inhibition, red arrow: schematic DNA segment.

In tumors responsive to anti-estrogen, the chief action against AF2 blockade is the restoration and amplification of the estrogen activation of ERs [56]:

1. Tamoxifen treatment provokes compensatory unliganded ER activation without delay by ER-alpha translocation from the nucleus to the membrane-bound EGFRs [179] (Figure 1); 2. The long term “therapeutic” ER blockade amplifies the expression of the ER-alpha coactivator; AIB1 (amplified in breast cancer 1) [180]. Under tamoxifen treatment, another coactivator of ERs, cyclin D1 amplifies the activation of both STAT3 and ERs [181]; 3. Tamoxifen treatment highly activates the transcription factor NFκB and its upregulative interaction with ER-alpha [182,183]; 4. Tamoxifen induces the increasing expression of certain microRNAs that bind to ER mRNAs, activating the translational processes [184]; 5. Tamoxifen provokes the amplification of the *ESR1* gene associated with the increased expression and activation of ERs [185,186] (Figure 2); 6. Aromatase inhibitor treatment provokes an acquired amplification of the *CYP19A1* gene, increasing both aromatase expression and estrogen synthesis [187]; 7. In tumor cells treated with tamoxifen, abundant lncRNA transcripts of ERs mediate the activating mutations for crucial genes of the genome stabilizer circuit; such as *ESR1*, *BRCA1*, and *CYP19A* [56].

### 8.2. Unsuccessful Fight of Tumors Non Responsive to Endocrine Disruptor Treatment

**Figure 3:**
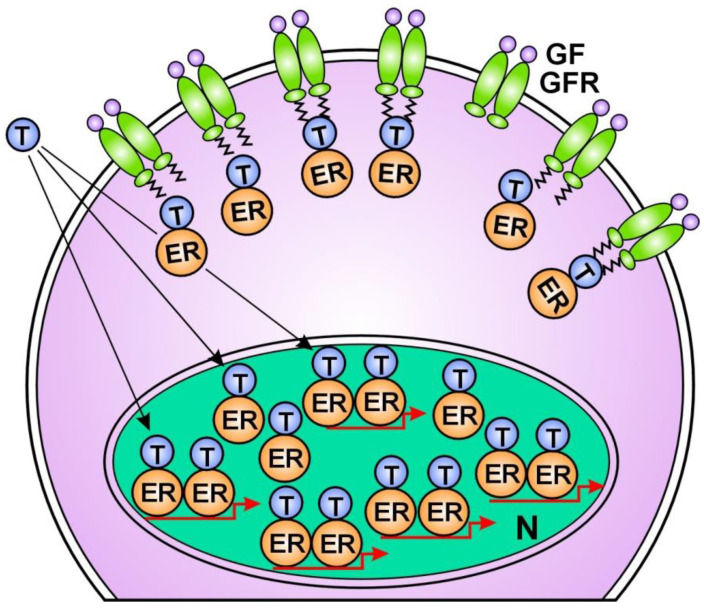
Molecular mechanism of tumor resistance in Tamoxifen (T)-treated cancer cells. The liganded activation of abundant nuclear estrogen receptors (ERs) is completely blocked by T-binding. The compensatory abundant expression of membrane-associated growth factor receptors (GFRs) struggles for the unliganded activation of T-bound ERs. However, the T blockade inhibits the restoration of ER signaling resulting in unrestrained proliferation. GF: growth factor, N: nucleus, black solid arrow: inhibition, spiral: unsuccessful activation, red arrow: schematic DNA segment.

In anti-estrogen responsive breast cancers, the increased regulatory processes promote the compensatory improvement of estrogen activation of ERs and may achieve a successful tumor response [188]. Earlier anti-estrogen responsive breast cancers become non-responsive as the possibilities for liganded ER activation are exhausted. In non-responsive tumors, increased growth factor receptor signaling remains an ultimate refuge for unliganded ER activation and DNA stabilization [17]. However, when the liganded ER activation is completely blocked, the increased unliganded activation of ERs is incapable of restoring ER signaling (Figure 3).

In anti-estrogen resistant breast cancers, physiological regulatory pathways are working so as to increase unliganded ER activation. In tamoxifen-resistant cancers, the ER coactivator HOXB7 exhibits an increased expression and may activate kinase phosphorylation of both EGFR [189] and HER2 [190], promoting unliganded ER activation. Further ER coactivators—AIB1 and HER2/neu—stimulate hormone-free ER activation [191]. In tumor xenografts, both ER and HER2 activations were coupled with the compensatory activation of MUCIN4 [192]. In anti-estrogen resistant tumors, the increased expressions of plasma membrane-bound EGFRs [193] and IGF-1Rs [194,195] amplify unliganded ER activation. In endocrine-resistant cancers, acquired somatic mutations may strongly increase the compensatory hormone-free ER activation.

In tumors resistant to endocrine therapy, acquired somatic mutations may strongly increase the compensatory hormone-free activation of ERs:

1. Estrogen conferred somatic mutation of *ERBB2* gene amplifies the expression and activity of growth factor receptors, conferring estrogen-free ER activation [191]; 2. In endocrine refractory ER-positive breast tumors, the *PIK3CA* gene is frequently mutated, upregulating the components of the PI3K-AKT-mTOR pathway and increasing hormone free ER activation [196]; 3. In AI-resistant breast cancers, acquired point mutations in the ligand binding domain (LBD) of *ESR1* gene confer hormone-independent activation of ERs [142]; 4. In anti-estrogen resistant tumors, chromosomal rearrangement on the *ESR1* gene leads to somatic mutations driving an increased unliganded activation of ERs [144]; 5. In tamoxifen-resistant tumor cells, the activation of the PI3K/AKT pathway led to a significant increase in BARD1 and BRCA1 protein expressions via increased estrogen independent activation of ERs [197].

## 9. Estrogen Induced Apoptosis Is Promising in Both the Prevention and Therapy of Cancer

Estrogen treatment of breast cancers resistant to either long term estrogen deprivation (LTED-R) or tamoxifen (TAM-R) triggers an apoptotic death in tumors [198].

In clinical practice, estrogen dramatically decreased the mortality of advanced breast cancer cases after stopping the long term tamoxifen therapy [199]. Following long term estrogen deprivation, estrogen reduced metastatic tumors and prolonged the survival of patients [200]. The biology of estrogen-induced apoptosis in breast and prostatic cancers seem to be promising in both the prevention and therapy of tumors [201].

Breast cancers unresponsive to anti-estrogen treatment exhibit extreme upregulation of both ER and GFR expressions. Estrogen may exert intensive anti-cancer capacity via balanced liganded and unliganded activation of abundant ERs. In reality, estrogen treatment does not return non-responsive tumors to anti-estrogen sensitivity. Conversely, estrogen helps tumor cells to defeat the genotoxic drug as they are highly sensitized to estrogen signal.

Important lessons may be drawn from the 50 years of breast cancer therapy with anti-estrogens: 1. In tumors, there is no endocrine therapy resistance, but rather the possibilities for compensatory ER activation are exhausted; 2. In tumors responsive to anti-estrogen therapy, increased ER expression and activation is not a survival technique, but rather it is an effort for increasing estrogen signaling; 3. In tumors non-responsive to anti-estrogen therapy, increased growth factor receptor expression and activation is not a survival technique, but rather it is an effort for compensatory unliganded ER activation; 4. Tumors exhaustively treated by aromatase inhibitors, show genomic plasticity, exhibiting acquired mutations on the ligand binding domain of *ESR1* gene conferring new, hormone-independent activation of modified ERs in the absence of estrogen.

## 10. Conclusions

Compared to various organs, female breasts exhibit unique sensitivity to genomic instability caused by either germline or acquired gene mutations. This fact may partially explain why breast cancer has become the flagship of cancer research. Although the preconception of “estrogen-induced” breast cancer has led breast cancer care to a quite erroneous pathway, a thorough examination of the controversies between estrogen signaling and cancer development yielded valuable progress in overall cancer research.

The correlation between genomic instability and conspicuously increased breast cancer risk in germline *BRCA* gene mutation carriers revealed that the defect in the genome stabilizer circuit is the origin of cancer initiation, rather than excessive estrogen signaling. Defects in ER, BRCA, or the aromatase enzyme upsets the triangular partnership of these regulatory proteins, leading to weaknesses in estrogen signaling and genomic instability. *BRCA* mutation carrier healthy and tumor cells similarly show efforts for increasing the liganded and unliganded ER activation and for compensatory upregulation of another genome safeguarding protein, p53.

Understanding the fight of cancer cells for the activation of estrogen signaling, together with genome stabilization, reveals the secret of various receptor landscapes of breast cancer subtypes. In tumors, the increased expression of hormone receptors reflects efforts for increasing liganded ER activation, while the overexpression of HER2 represents trying to increase unliganded ER activation. The blockade of either ERs or HER2s seems to be an erroneous therapeutic concept. Breast cancers are not resistant to genotoxic therapies, but rather they exhausted all possibilities for defending the remnants of genomic stability. Progressive genomic instability leads to unrestrained proliferative activity.

The cellular infiltration of the tumor microenvironment is not an organic part of tumors. Inflammatory cells are recruited by the tumor itself and the intercellular communication by messages and extracellular vesicles confer in asking for help. The stronger the genomic deregulation in the tumor, the denser is the adjacent infiltration of activated mesenchymal and immune competent cells. Immune competent cells do not need therapeutic genomic machination as they know exactly their task in the anti-cancer fight. When tumor invasion is coupled with dense peritumoral infiltration, supportive genome repairing therapy is necessary, rather than the disruption of mutation-activated DNA repair pathways of tumors.

In conclusion, the improvement of genomic stability may be the new strategy in cancer therapy. The upregulation of estrogen signaling leads to strengthened immune response, whilst inducing the apoptotic death of tumors in a Janus-faced manner.

## Figures and Tables

**Table 1:** Receptor pattern in breast cancer subtypes reflecting the grade of DNA damage and the concomitant actions for DNA repair.

Subtype of Breast Cancer	Receptor Status	Signs of DNA Damage	Sigs of DNA Repair	Proliferative Activity Endocrine	Response to Therapy
Luminal A type	ER overexpression	no	ER overexpression	low	good in 50%
(50–60%)	PR positive				
Luminal B type	ER positive	PR negative	ER positive	increased	moderate/inverse
(10%)	PR pos/neg	PR positive			
	HER2 pos/neg	HER2 positive			
HER2 enriched	ER negative	ER negative	HER2 rich	high	no
(20%)	PR negative	PR negative			
	HER2 rich				
Triple negative	ER negative	ER negative	no	high	no
(10%)	PR negative	PR negative			
	HER2 negative	HER2 negative			
